# Food Safety in Informal Markets: How Knowledge and Attitudes Influence Vendor Practices in Namibia

**DOI:** 10.3390/ijerph22040631

**Published:** 2025-04-17

**Authors:** Winnie L. N. Sheehama, Tanusha Singh

**Affiliations:** Department of Environmental Health, University of Johannesburg, Johannesburg 2038, Gauteng, South Africa; shlesian2@gmail.com

**Keywords:** food safety, food handlers, informal markets, public health, food safety training, KAP, foodborne diseases, street food

## Abstract

**Background**: Informal markets are essential to ensuring food accessibility and supporting economic livelihoods throughout sub-Saharan Africa; however, food safety in these settings remains poorly regulated and under-resourced. As such, foodborne illnesses originating from such settings pose serious public health threats. Despite the growing reliance on ready-to-eat street foods in Namibia, little is known about the food safety knowledge, attitudes, and practices (KAP) of food handlers in informal settings. This study investigated food handlers’ KAP regarding food safety at the Oshakati Mini Market, northern Namibia, to inform environmental health practitioners and guide policymakers in improving food safety measures. **Methods**: A cross-sectional study was conducted with 103 food handlers using a self-administered questionnaire which assessed sociodemographic characteristics, food safety knowledge (15 items), attitudes (10 items), and practices (12 items). Data were analysed using descriptive statistics, Pearson correlation, and multiple linear regression to identify associations between KAP scores and respondent characteristics. **Results**: The majority of respondents were female (60.2%), aged 30–49 years (69.0%), and had only primary-level education. Only 15.5% had received formal food safety training. The mean knowledge score was 64.7% (SD = 14.2), and the mean practice score was 58.2% (SD = 13.8). Attitudes were predominantly negative (60.2%), despite 95.1% acknowledging the importance of handwashing. Poor practices included low use of protective clothing (28.2%), smoking in food areas (21.4%), and inadequate utensil hygiene. Knowledge was positively correlated with good practices (r = 0.745, *p* < 0.01), while attitudes were negatively correlated with good practices (r = −0.745, *p* < 0.01). Regression analysis revealed that age negatively influenced knowledge (β = −0.265, *p* < 0.01), while work experience positively predicted both knowledge (β = 0.393, *p* < 0.01) and practices (β = 0.393, *p* < 0.01). **Conclusions**: Food handlers in Oshakati’s informal market exhibited moderate knowledge but sub-optimal food safety practices and largely negative attitudes. Limited training and education were key contributing factors. These findings highlight an urgent need for structured, context-specific food safety training and regulatory enforcement to reduce foodborne disease risk and strengthen public health interventions in Namibia’s informal food economy.

## 1. Introduction

Informal food markets are a vital component of food systems in low- and middle-income countries (LMICs), providing affordable and accessible meals to urban and peri-urban populations [1]. They provide essential employment and income-generating opportunities, serving as primary sources of livelihood for women, migrants, and low-income households [2,3]. Food handlers in such environments play a critical role in either mitigating or amplifying food safety risks, depending on their knowledge, attitudes, and practices (KAP). While many studies do not directly examine KAP in these markets, they highlight the vendors’ resilience and innovation in adapting to challenges such as limited access to finance, competition, and evolving consumer needs. In Cape Town, South Africa, informal food vendors are central to food flows, but their exclusion from regulatory frameworks, especially during crises like COVID-19, has led to significant disruptions, food insecurity, and job losses [4,5]. Similarly, in the Kathmandu Valley, informal food markets are vital for poverty alleviation but face threats from municipal authorities, calling for improved governance to support urban livelihoods [3]. In Mexican cities, these markets enhance the accessibility and affordability of fresh foods for low-income groups [6]; however, a major concern across the informal food systems is poor food safety practices. Research from Dhaka and Jordan reveals that while vendors may have adequate knowledge or positive attitudes, actual food handling practices are often unsafe, increasing the risk of foodborne illnesses [7]. Despite their significance, informal food markets face numerous challenges, often characterised by poor infrastructure, regulatory neglect, competition, substandard hygiene practices, among other health and safety concerns [8], making them hotspots for foodborne disease transmission. Varying levels of knowledge, attitudes, and practices among food handlers have been reported across different regions, often influenced by sociodemographic factors and the availability of resources [1,9].

Food handlers play a crucial role in upholding food safety standards, as they can inadvertently transmit harmful microorganisms across food processing, distribution, and consumption stages [10,11,12,13]. Globally, foodborne diseases affect one in ten people annually, resulting in 420,000 deaths and approximately 34 million disability-adjusted life years (DALYs), with the economic burden in LMICs estimated at USD 121 billion. Africa bears the highest burden of foodborne illnesses, with 91 million cases and 137,000 deaths estimated annually [10]. A significant portion of relatively inexpensive food purchased and consumed in Africa is sourced from informal markets that often operate without adequate regulation [14,15]. With increasing urbanisation and the demand for food on the go, foods sold in these unregulated informal markets may increase the risk of foodborne illness along the food value chain [14]. Despite the known risks, research on the behavioural and structural drivers of food safety in informal markets remains limited in sub-Saharan Africa. Studies from South Africa highlighted that food vendors are integral to the food preparation and distribution process, contributing to foodborne illnesses [16,17]. The absence of effective preventative food safety measures exacerbates concerns about food handlers’ KAPs in this market. In LMIC like Namibia, foodborne diseases affect more than a third of the population, primarily due to food purchased from informal market vendors [18,19]. Yet, little research has focused on food safety knowledge, attitudes, and practices (KAP) in these settings. This contrasts with findings from an Indonesian study, which showed that food handlers at such markets demonstrate insufficient KAPs concerning food safety [20]. Unlike other countries in the region, where studies have explored food handlers’ KAPs, Namibia has a notable research gap in this area. The open-market food handlers in Namibia remain an understudied group; therefore, assessing their food safety knowledge, attitudes, and practices and their association with sociodemographic factors can provide regulatory bodies with the necessary data to develop evidence-based interventions, ensuring safer food for consumers. Gaps in knowledge, negative attitudes, and unsafe practices directly affect public health. Assessing KAP helps identify critical weaknesses and informs targeted interventions, enabling policymakers and environmental health practitioners to strengthen food safety standards, reduce disease risk, and support safer, more sustainable informal food systems. This study addresses these gaps by investigating the food safety KAP of food handlers in a major informal market in northern Namibia. By applying a structured KAP model and linking it to sociodemographic predictors, the study offers novel insights into the behavioural drivers of food safety compliance. This research not only provides baseline data for policymakers and environmental health practitioners but also proposes evidence-based recommendations tailored to local realities. This is, to our knowledge, one of the first studies in Namibia to rigorously analyse the determinants of food safety behaviour in informal urban markets using both descriptive and multivariate methods.

## 2. Materials and Methods

This study employed a descriptive cross-sectional design, (Kothari 2004) guided by the Strengthening the Reporting of Observational Studies in Epidemiology (STROBE) guidelines, and was conducted at the Oshakati Mini Market in Oshakati, Namibia’s second-largest town [21]. The STROBE model was intentionally selected to ensure methodological rigour and comprehensive reporting of the research process, providing a structured framework for improving the quality and replicability of the research. The market comprises approximately 800 stalls, offering a variety of goods, including traditional foods, cooked meals, kapana (braai meat), tailoring services, cosmetics, produce, clothing, and cell phone repairs. The market is divided into three sections (A, B, and C), each housing food handler booths.

The target population included 130 food handlers selling Western food, kapana, and traditional dishes, who were conveniently sampled. Eligible respondents were 18 years or older, had at least one year of experience at the Oshakati Mini Market, and were actively engaged in food handling. This employment duration was deemed sufficient to ensure familiarity with food safety knowledge, attitudes, and practices. Food handlers with less than one year of experience and those under 18 years were excluded from the study. A convenience sampling strategy was employed to select respondents. The sample size was determined using Taro Yamane’s formula (n = N ÷ (1 + Ne^2^), where N represents the study population, *n* the sample size, and *e* the margin of error, set at 5%. Based on a total population of 130 food handlers, the calculated sample size was 98 at a 95% confidence level. To account for potential non-responses, a 5% contingency was added, bringing the final sample size to 103 participants. Eligibility criteria included individuals employed as food handlers at the Oshakati Mini Market, aged 18 years or older, with at least one year of experience. This duration was deemed sufficient to ensure familiarity with food safety knowledge, attitudes, and practices. Data collection was conducted over a one-month period, from 1 April to 30 April 2024.

The research strategy involved administering a self-administered, closed-ended questionnaire (Supplementary Materials S1), adapted from previous studies [11,22,23,24] to align with the study objectives. The questionnaire was piloted on ten individuals, who were later excluded from the main study. The pilot study produced a Cronbach’s alpha (α) value exceeding 0.7, indicating that the instrument was both reliable and consistent. Each respondent took approximately 15–20 min to complete the survey. The questionnaire was administered in English, as it is the official language taught in schools and understood by over 60% of Namibians. However, an interpreter was available to translate the content into Oshiwambo when needed. The questionnaire consisted of three key sections as shown in Table 1.

The data were entered, cleaned, and coded in Microsoft Excel (Microsoft Corporation, Redmond, WA, USA). Incomplete surveys were removed before being exported to SPSS version 29.0 (IBM Corp., Armonk, NY, USA) for analysis [25]. To ensure data quality and completeness, multiple validation checks were performed at each stage. However, for minimal missing data, mean imputation was applied to continuous variables, reducing data loss while maintaining the study’s statistical power. The dataset included respondents’ sociodemographics, knowledge, attitudes, and practices (KAP) related to food safety. Factors such as age, education level, work experience, and training play a critical role in shaping food handlers’ food safety knowledge and practices. Descriptive statistics (frequencies and percentages) were used to summarise the demographic characteristics of respondents and the distribution of KAP related to food safety. Categorical variables were summarised using frequencies and percentages, whereas continuous variables like age and work experience were reported as means with corresponding standard deviations and ranges, where applicable. Composite indices were developed for each KAP domain, where knowledge scores were based on the number of positive responses, with scores ≥ 50% classified as “adequate” knowledge; attitudes were categorised as positive or negative based on “Yes” responses; and practices were deemed good if respondents consistently answered “Yes” to recommended food safety behaviours, and poor if they answered “Sometimes” or “No”. The scores were then categorised and analysed as binary outcomes (adequate/inadequate, positive/negative, good/poor) for further statistical testing. Inferential statistics were applied to examine relationships between sociodemographic variables (including age, education, marital status, sex, work experience, and training) and food safety KAP. Pearson correlation analysis was performed to assess the strength and direction of relationships between continuous or ordinal variables. Multiple linear regression analyses were conducted for each KAP domain to identify significant sociodemographic predictors while adjusting for potential confounding factors. The results were presented as β coefficients, *p*-values, R^2^, and adjusted R^2^, with statistical significance set at *p* < 0.05.

## 3. Results

### 3.1. Demographic Characteristics of the Study Respondents

In this study, 130 questionnaires were distributed to food handlers at the Oshakati Open Market, of which 103 completed questionnaires were returned, resulting in a 79.2% response rate. Table 2 delineates the demographic characteristics of the food handlers who participated in the study. There were more females (62, 60.2%) than males (41, 39.8%) and the most dominant age group was 40–49 years (36, 35.0%), closely followed by 30–39 years (35, 34%), with the fewest in the group 18–20 years (4, 3.9%). Most study respondents were single (59, 57.3%) and a quarter were married (26, 25.3%). Many of the food handlers (39, 37.9%) had a primary education, followed by those with secondary education (34, 33.0%) and without formal education (21, 20.4%). Most respondents’ working experience ranged from 4–10 years (39, 37.9%), followed by 11–15 years (24, 23.3%).

### 3.2. Food Handlers Trained in Food Safety

Less than a fifth of the food handlers (15.5%) received formal training on food safety compared to 84.5% who did not receive any training (Table 3). Most training was obtained in Oshakati (second largest city) (8, 50%), followed by Windhoek (capital city) (6, 37.5%) and Otjiwarongo town (2, 12.5%). Most respondents were trained in 2022 (7, 43.8%), and the fewest trained before 2020.

### 3.3. Food Handlers’ Level of Knowledge, Attitudes, and Practices on Food Safety

**Figure 1:**
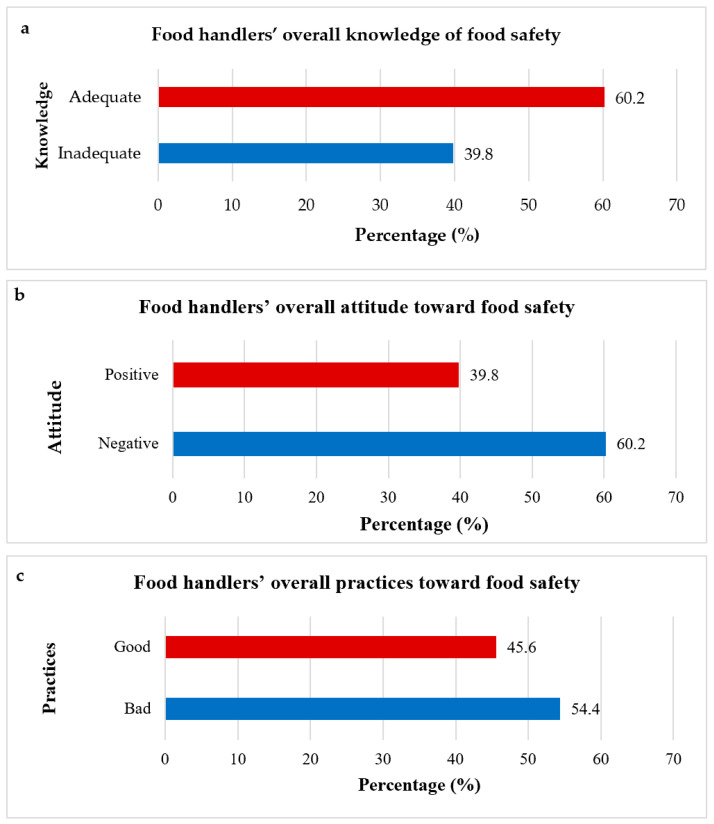
(**a**) Food handlers’ overall knowledge of food safety, (**b**) food handlers’ attitudes toward food safety, and (**c**) food handlers’ practices on food safety.

Using the average Likert scale scores, the food handlers’ level of knowledge on food safety was assessed based on their general knowledge regarding food safety, knowledge regarding contamination and transmission of foodborne diseases, knowledge regarding temperature control and storage, knowledge regarding personal hygiene, and their overall knowledge regarding food safety. The food handler’s overall food safety knowledge was regarded as adequate or inadequate. Adequate food safety knowledge was determined by the respondents’ correct responses to the questions, while incorrect responses indicated inadequate food safety knowledge. As illustrated in Figure 1a, almost two-thirds (62, 60.2%) of the food handlers had adequate food safety knowledge compared to those (41, 39.8%) deemed to have inadequate food safety knowledge.

The responses to food handlers’ attitudes were categorised as either positive if respondents answered ‘yes’ or negative if they responded ‘no’ or ‘I do not know’. The overall distribution of food handlers’ attitudes towards food safety indicated that the majority (62, 60.2%) exhibited negative attitudes toward food safety, while 39.8% (*n* = 41) demonstrated positive attitudes, suggesting a need for intervention and education initiatives to improve food safety awareness among food handlers (Table 1).

Food handlers’ practices were assessed as good or bad based on the Likert scale score averages based on their responses to the questions posed regarding practices. Practices were considered good if respondents consistently answered ‘yes’, indicating regular adherence, while practices were deemed bad if respondents answered ‘sometimes’ or ‘no’. Slightly more food handlers (56, 54.4%) demonstrated bad practices, compared to those (47, 45.6%) that exhibited good practices in food safety (Figure 1c).

### 3.4. Correlation Between Food Handlers’ Sociodemographic Characteristics and Knowledge, Attitudes, and Practices

The Pearson correlation analysis revealed a strong positive correlation (r = 0.745, *p* < 0.01) (Table 4) between food safety knowledge and food safety practices, indicating that better knowledge is associated with improved food safety behaviours. In contrast, a strong negative correlation (r = −0.745, *p* < 0.01) was found between food safety attitudes and practices, suggesting that more positive attitudes toward food safety did not necessarily translate into better food handling practices. Additionally, work experience demonstrated a weak but significant correlation with attitudes (r = −0.229, *p* < 0.05) and knowledge (r = 0.229, *p* < 0.05), reinforcing its importance in shaping food handlers’ perceptions and behaviours. Furthermore, work experience showed significant associations with age (r = 0.605, *p* < 0.01) and education level (r = 0.467, *p* < 0.01), reflecting the cumulative nature of professional development over time. However, sex, marital status, and training did not significantly correlate with knowledge, attitudes, or practices, indicating that these factors may have minimal influence on food handlers’ food safety behaviours.

### 3.5. Multiple Linear Regression Analysis of Food Handlers’ Sociodemographic Characteristics and Knowledge, Attitudes, and Practices of Food Safety

Multiple linear regression analysis was employed to explore the extent and nature of the relationship between sociodemographic factors (including age, education level, marital status, sex, training, and work experience) and food handlers’ KAPs related to food safety. The regression model indicated a moderate correlation (R = 0.325) between sociodemographic predictors and food safety knowledge, explaining 10.6% of the variance (R^2^ = 0.106, Adjusted R^2^ = 0.050). However, the model was not statistically significant, (F (6, 96) = 1.893, *p* = 0.090), suggesting that sociodemographic factors alone do not strongly influence food handlers’ knowledge of food safety. A statistically significant negative relationship was found between age and knowledge (β = −0.265, *p* < 0.01), indicating that as age increases, knowledge of food safety decreases (Table 5). Conversely, work experience positively influenced knowledge (β = 0.393, *p* < 0.01), implying that more experienced food handlers possess better food safety knowledge. However, sex, marital status, education level, and training did not significantly predict food safety knowledge, attitudes, or practices.

For food safety attitudes, the model also showed a weak correlation (R^2^ = 0.208, Adjusted R^2^ = 0.062), indicating that 20.8% of the variance was explained by sociodemographic factors. Similar to knowledge, the model was not statistically significant (F (5, 92) = 2.967, *p* = 0.081), suggesting that attitudes are not strongly influenced by sociodemographic factors. Unlike the construct knowledge, age was positively correlated with attitudes toward food safety (β = 0.079, *p* < 0.01) (Table 5), suggesting that older food handlers have more favourable attitudes toward food safety. In addition, work experience negatively influenced attitudes (β = −0.162, *p* < 0.01), implying that more experienced food handlers possess poorer food safety attitudes.

In contrast, food safety practices were significantly predicted by sociodemographic factors, (F (7,95) = 17.6, *p* = 0.000), with a strong correlation (R = 0.751) and 56.5% variance explained (R^2^ = 0.565, Adjusted R^2^ = 0.532). This suggests that food handlers’ sociodemographic characteristics, particularly work experience, strongly influence their food safety practices. Regarding food safety practices, work experience was the only significant predictor (β = −0.014, *p* < 0.05) (Table 5), highlighting the critical role of experience in shaping food safety behaviours. Other variables, including age, sex, marital status, education level, and training, were not significant predictors of food safety practices.

## 4. Discussion

This study provides critical insights into the food safety knowledge, attitudes, and practices (KAP) of informal food vendors operating at the Oshakati Mini Market in northern Namibia. The findings reflect a broader concern common to many LIMCs; although informal markets serve vital socio-economic and nutritional roles, the poor alignment between KAPs continues to undermine food safety efforts and increase public health risks.

### 4.1. Demographic Characteristics of Respondents

The current study found that most food handlers at the Oshakati Open Market were female, aged 40–49 years, and had attained only primary education. These findings align with previous research by Moreb et al. (2017) and Vo et al. (2015), which also reported a predominance of women in the food service sector [26,27]. This demographic skew may explain some of the disparties in food safety behaviour, particularly when combined with limited training opportunities, a finding consistent with studies from Nigeria [28] and Vietnam [27,29], where food vendors had limited access to food safety education. The finding could also imply either a lack of effective training methodologies or the informal and unstructured nature of the training received. Without ongoing refresher sessions or reinforcement, knowledge gained during one-off sessions may be poorly retained or applied. Furthermore, the absence of training is concerning, as it contributes to poor food handling practices and increases the risk of foodborne illnesses. Additionally, the lack of food safety inspections at the Oshakati Open Market highlights gaps in regulatory enforcement, further compromising food safety compliance.

To improve food safety training and compliance in informal markets, a multi-tiered approach should be implemented. This includes mandatory, low-cost training programmes in local languages, mobile outreach and on-site training models, certification systems, refresher courses, community radio campaigns, and collaboration between local authorities, public health institutions, and vendor associations. These strategies aim to create a supportive regulatory framework that encourages self-monitoring, peer accountability, and regular inspection without being punitive. This approach can transform informal markets into safer environments for both vendors and consumers, and can be scaled nationally to reduce the public health burden of foodborne diseases.

### 4.2. Food Handlers’ Knowledge of Food Safety

Although 60.2% of participants demonstrated adequate food safety knowledge, significant gaps remained, particularly in understanding temperature control and safe storage practices, a proportion higher than the 31% and 47.5% reported by Safari et al. (2018) in Iran [30] and Siddiky et al. (2022) in Bangladesh [31], respectively, but lower than 76% and 90% found by Ahmed et al. (2021) in Pakistan [32] and Osaili et al. (2018) [33] among hospital and restaurant food service staff [33]. Knowledge of temperature control and storage was particularly weak, with more than half (58.1%) unaware of the legal temperature requirements for heating, chilling, and freezing food. Similar gaps have been reported by Farhana et al. (2020), where only 28% of food handlers demonstrated correct food storage practices [24]. Poor temperature control facilitates the growth of foodborne pathogens, posing a serious public health risk in informal food markets [34]. Food handlers in this study demonstrated better knowledge of contamination prevention (59.5%) and personal hygiene (66.3%), particularly in handwashing practices. These findings are consistent with Addo-Tham et al. (2020) in Ghana [10] and Ahmed et al. (2021) in Pakistan [32], who also reported high levels of hygiene awareness among food handlers. Notably, food handlers in the current study were more knowledgeable about personal hygiene, especially handwashing, than they were about contamination risks or storage practices. This selective knowledge profile suggests that public health messaging around hygiene may be partially effective, but comprehensive food safety literacy remains insufficient. Despite the moderate overall knowledge, deficiencies in temperature control and food storage remain critical concerns, highlighting the need for targeted training interventions. Studies examining food safety KAP among street vendors reveal regional variations. In Chennai, many street vendors lack formal knowledge, particularly in personal hygiene and food handling [35]. In Nigeria, 67% of food vendors demonstrated good knowledge of food hygiene and safety [36], whereas in Jordan, vendors exhibited moderate knowledge but had gaps in understanding specific pathogens [5]. Similarly, in Dhaka, 62% of vendors had adequate food safety knowledge, although most lacked formal training [7]. These discrepancies suggest that food safety knowledge varies significantly across different settings, often depending on education, training availability, and regulatory oversight. Notably, only 15.5% of food handlers in this study had received formal training, a lower proportion than in studies conducted in South Africa (Letuka et al., 2021) [11] and Indonesia (Putri and Susanna, 2021) [20], where ongoing vendor education programmes were in place.

One critical myth identified in the food handlers’ knowledge about food safety was the misconception that the taste or smell of food is a reliable indicator of its safety. Several respondents believed that as long as food tasted or smelled acceptable, it was safe for consumption. This belief is problematic, as many foodborne pathogens do not alter the organoleptic properties of food, meaning contaminated food can look, smell, and taste normal while still posing serious health risks. This myth reflects a significant gap in scientific understanding of microbial contamination and highlights the urgent need for targeted educational interventions that address such misconceptions among informal market food handlers.

### 4.3. Food Handlers’ Attitudes Toward Food Safety

Contrary to findings from Indonesia and South Africa (Putri and Susanna, 2021; Letuka et al., 2021), this study revealed that the majority of food handlers in Oshakati (60.2%) held negative attitudes toward food safety [11,20]. This is particularly concerning given the critical role of attitudes in shaping motivation and compliance behaviour. Although 72.8% of respondents expressed a willingness to learn about food safety, suggesting that interventions could effectively shift attitudes, 70.9% did not undergo regular medical check-ups, and only 28.2% wore uniforms during food preparation, indicating low adherence to hygiene regulations. This contrasts with Letuka et al. (2021), who found that over half of the respondents believed protective gear was essential in preventing food contamination [11]. The observed negative attitudes may reduce motivation for food safety compliance, heightening the risk of foodborne disease outbreaks. This poses a significant public health concern, particularly in Oshakati, Namibia, where many people rely on informal food vending for their daily sustenance. The divergence between studies likely reflects structural differences between formal and informal food systems, with informal vendors often lacking external accountability and formal oversight.

### 4.4. Food Handlers’ Food Safety Practices

Despite moderate knowledge scores, food safety practices among respondents were generally poor, with only 45.6% classified as having good practices, a concerning finding that contradicts that from Farhana et al. (2020) and Kundu et al. (2021), who reported 88.82% and 83.1% compliance rates in Bangladesh [24] and India [37], respectively. Key deficiencies included low adherence to hygiene practices, such as wearing protective clothing (46.6%), using detergents for cleaning utensils (29.1%), washing hands regularly (27.0%), and keeping long nails (56.3%). These poor practices suggest that moderate food safety knowledge does not necessarily translate into proper application. These behaviours indicate breakdowns in both personal hygiene and environmental health compliance, with potential implications for microbial contamination of ready-to-eat foods. The findings are consistent with those from Ghana [10] Bangladesh [38], and Jordan [5] where knowledge was not always predictive of safe practices. These comparisons between different countries show that while food safety issues are prevalent across many developing countries, the severity and type of challenges differ based on contextual factors like education access, infrastructure, and regulatory presence. While only 39.8% of food handlers demonstrated positive attitudes toward food safety, a slightly higher proportion (45.6%) reported engaging in good food safety practices. This finding suggests that while many food handlers may not internalise or endorse food safety principles at the attitudinal level, some still follow acceptable practices, possibly due to external influences such as peer norms, customer expectations, or fear of regulatory consequences. It could also indicate habitual behaviour formed over time, even in the absence of strong attitudinal alignment. Importantly, these findings reinforce the “knowledge–behaviour gap” commonly observed in food safety literature. Economic pressures, informal work conditions, and cultural norms may prevent food handlers from applying even the knowledge they possess. The similarity in poor practices despite moderate knowledge, as also observed in Farhana et al. (2020) [24], reinforces the idea that knowledge alone is insufficient to guarantee safe food handling. Economic pressures, limited access to resources, and ingrained cultural norms may inhibit behaviour change even among knowledgeable handlers. The negative correlation between attitudes and practices (r = −0.745) in our study further supports this theory, showing that even when handlers report positive beliefs, their actual practices may not align, especially in unregulated environments.

### 4.5. Interrelationship Between Knowledge, Attitudes, and Practices

A particularly striking finding was the inverse relationship between positive attitudes and safe practices (r = −0.745, *p* < 0.01), indicating a disconnection between the perceived importance of food safety and actual behaviour. This suggests that although food handlers may intellectually grasp food safety principles, this understanding does not always translate into motivation or consistent practice. Contributing factors may include weak regulatory enforcement, the informal nature of their work, and the absence of meaningful incentives to comply. This observation aligns with the Health Belief Model, which emphasises that perceived risk and barriers can hinder the translation of knowledge into action. The significant positive correlation between knowledge and practice scores indicates that respondents who demonstrated higher levels of knowledge were more likely to engage in safer food handling behaviours. This relationship suggests that food safety knowledge is a critical determinant of actual practice among food handlers in informal markets.

In contrast, the study found a strong positive correlation between knowledge and safe food handling practices (r = 0.745, *p* < 0.01), suggesting that greater food safety knowledge significantly enhances practice. This supports the KAP model (Beauregard et al., 2019) [39], which posits that knowledge fosters attitudinal change that can lead to improved behaviour. Additionally, knowledge was highly correlated with attitudes (r = 1, *p* < 0.01), reinforcing the role of education in shaping food safety perceptions.

Negative attitudes may reinforce complacency, reducing adherence to food safety guidelines. Despite recognising the importance of hand hygiene, food handlers showed low compliance with other hygiene measures, such as wearing protective clothing or properly handling food, indicating gaps between knowledge and implementation. This finding is consistent with previous research by Hassan et al. (2017) [38], which showed that vendors often demonstrate awareness but engage in risky behaviours due to structural or contextual challenges.

### 4.6. Relationship Between Sociodemographic Characteristics and Food Safety KAP

The regression analysis highlighted work experience as a key factor influencing both knowledge and practices (β = 0.393, *p* < 0.01 and β = −0.014, *p* < 0.05, respectively). This highlights the potential for informal learning and adaptation over time, despite a lack of formal training, reinforcing that longer exposure to food handling improves adherence to food safety behaviours. This finding is consistent with Siddiky et al. (2022), who noted that experienced food vendors learned from past mistakes [31], allowing them to implement better risk mitigation strategies. The finding also suggests that peer-to-peer mentoring, especially from experienced vendors, could serve as a low-cost strategy for disseminating practical food safety skills. Conversely, the negative association between age and knowledge (β = −0.265, *p* < 0.01) suggests a generational knowledge gap, with older food handlers less exposed to evolving food safety concepts and technologies, supporting findings by Siddiky et al. (2022) [31], who observed that older food vendors had better food safety attitudes. The finding also suggests that experience fosters greater awareness of foodborne risks, highlighting the importance of peer mentoring to enhance knowledge transfer among less-experienced food handlers. These findings highlight the critical role of practical experience in shaping food safety practices in informal markets and suggest that age-related knowledge gaps could be addressed through structured peer-to-peer mentoring and targeted training interventions that leverage the experiential strengths of seasoned food handlers.

### 4.7. Policy Implications

The study’s findings highlight a pressing need to strengthen food safety governance in informal markets. Persistent knowledge gaps and unsafe practices among food vendors call for the development and enforcement of tailored regulations that prioritise high-risk areas, such as temperature control, hygiene compliance, and cross-contamination prevention. Policymakers should implement context-specific training frameworks, ensure equitable access to food safety education, and introduce incentive structures, such as vendor recognition schemes or rental discounts to encourage compliance. Additionally, the lack of formal oversight points to a broader regulatory gap; strategies must include provisions for structured inspections, streamlined food safety certifications, and better integration of informal vendors into urban food policy.

### 4.8. Public Health Implications

From a public health perspective, the study underscores the vulnerability of informal markets to foodborne disease transmission. Namibia’s burden of foodborne illness—particularly diarrhoeal diseases caused by *Salmonella* spp., *Escherichia coli*, and *Shigella* spp.—is likely exacerbated by the poor food handling practices observed in this study [1]. Given Namibia’s documented burden of foodborne disease, the informal food sector could represent a key node in the transmission pathway [8]. For instance, the study found that only 28.2% of food handlers wore protective uniforms, while 27.0% regularly washed their hands during food preparation. Furthermore, poor utensil cleaning practices (with only 29.1% using detergents) and the common habit of handling food and money, conditions that directly increase risk, have been reported. These failures in food safety practices align with key risk factors identified in outbreak investigations globally and regionally. The findings emphasise critical gaps in food safety knowledge and attitudes, emphasising the role of structured training programmes in preventing foodborne illnesses. While this study focused on individual KAP indicators, it also sheds light on structural weaknesses. The lack of regular health inspections, limited access to clean water, and overcrowded vending spaces all contribute to unsafe food environments. These issues reflect broader governance and resource allocation gaps that must be addressed if public health improvements are to be sustained. Environmental health practitioners must adopt a proactive approach, integrating health education, on-site mentorship, and real-time compliance checks into community engagement efforts. The importance of these findings is heightened by Namibia’s growing urban population and reliance on informal food vendors for daily meals. Furthermore, with Namibia experiencing a growing burden of foodborne illness, especially diarrhoeal diseases caused by *Salmonella* spp., *E. coli*, and *Campylobacter* [40], the gaps identified in temperature control, hygiene, and food storage underscore key points of vulnerability. By addressing these practices through tailored interventions, there is an opportunity to mitigate a significant portion of preventable disease burden.

### 4.9. Strengths and Limitations of the Study

This study effectively measured food handlers’ KAPs regarding food safety and examined how sociodemographic factors influenced these aspects. This provided valuable insights into food safety gaps and areas requiring improvement. The quantitative approach may not fully capture food handlers’ experiences; qualitative interviews could provide deeper insights. In addition, the study relied on self-reported data, which may be affected by recall bias and respondent honesty. As the study was conducted in a single open market, findings may not be generalisable to other Namibian markets.

What distinguishes this study is its Namibian setting, a country where informal food vendors contribute significantly to the urban food economy but remain underrepresented in academic research. Our findings provide the first empirical data on the KAP of open-market vendors in northern Namibia and offer a foundational baseline for future food safety interventions.

### 4.10. Recommendations

Based on the current study’s findings, several targeted actions are recommended to improve food safety in informal market settings such as the Oshakati Mini Market.

- Implement structured, context-specific food safety training tailored to local food handlers’ education levels and daily challenges. Training content should prioritise identified gaps, particularly in temperature control, food storage, and personal hygiene practices.
- Introduce behaviour change interventions that address the disconnect between knowledge and attitudes observed in the study. Interactive sessions, peer role-modelling, and incentive-based learning could improve motivation and translate knowledge into safer practices.
- Establish routine and visible food safety inspections, supported by local health authorities, to reinforce compliance. Inspections should focus on areas highlighted in the study as weak, including the use of protective clothing, handwashing, and utensil hygiene.
- Create informal vendor certification programmes. Vendors who demonstrate improved food safety practices could be prioritised for better stall locations or reduced rental fees, offering a positive incentive structure.
- Promote continuous learning by conducting refresher training and community-based health education campaigns through radio, posters, and trade fairs, targeting the majority of vendors who expressed a willingness to learn.
- Leverage experienced food handlers as peer educators, since the study showed that work experience positively influenced knowledge and practices. Peer-led training could bridge generational knowledge gaps and foster a culture of compliance.

### 4.11. Future Research

Future research should incorporate qualitative methods to explore the personal experiences, motivations, and perceptions underlying food handlers’ KAPs. This approach would provide deeper insights beyond quantitative findings, offering a more comprehensive understanding of factors influencing food safety behaviour in the region. Additionally, post-training intervention studies are needed to evaluate how enhanced knowledge influences attitudes and practices, ensuring the effectiveness of food safety training programmes. Future studies should also consider incorporating the type of food sold, such as Western food, kapana, and traditional dishes, as a variable of analysis when assessing food safety KAP. Disaggregating food handlers based on the categories of food they prepare and serve could reveal important variations in risk levels and hygiene behaviours associated with different types of cuisine. This would allow researchers and policymakers to design more tailored interventions and targeted training programmes that address specific food safety challenges unique to each category. Including this variable in future research could also improve our understanding of context-specific food handling practices within Namibia’s informal food sector.

## 5. Conclusions

This study revealed significant gaps in the knowledge, attitudes, and practices (KAP) of food handlers at the Oshakati Mini Market, a key informal food hub in northern Namibia. While a majority of food handlers demonstrated adequate knowledge of food safety, positive attitudes and consistent adherence to safe practices were lacking. This discrepancy highlights a critical knowledge-practice gap, posing substantial risks to public health, particularly in contexts where regulatory oversight is weak or inconsistent. The positive correlations between work experience and both knowledge and practices suggest that informal, experience-based learning may partially compensate for the lack of formal training. However, the very low percentage of trained vendors signals a systemic gap in access to structured food safety education. Age also emerged as a complex factor, with older food handlers expressing more positive attitudes but demonstrating lower levels of knowledge, underscoring the need for age-sensitive, ongoing training initiatives that are contextually relevant.

The lack of training, infrequent medical screening, inadequate hygiene practices, and negative attitudes observed in this study reflect systemic shortcomings in both health education and enforcement mechanisms. Informal markets are vital for food access and employment, yet without adequate regulation and support, they risk becoming persistent sources of foodborne disease outbreaks. The persistent mismatch between food safety knowledge and practice is especially concerning, as unsafe behaviours continue despite awareness. Given the rising burden of foodborne illnesses in Namibia, empowering food handlers through accessible training, improved infrastructure, and regulatory support is essential, not only to safeguard public health but also to strengthen the resilience and sustainability of urban food systems.

## Figures and Tables

**Table 1:** Structure of the self-administered questionnaire.

Domain	Sub-Domain	Number of Items
Knowledge	General knowledge, food contamination, hygiene, storage and temperature	12
Attitudes	Food safety perception, willingness to comply	7
Practices	Hygiene behaviours, food handling, cleanliness	10

**Table 2:** Demographic characteristics of respondents (*n* = 103).

Demographic Variable	Characteristic	Frequency (*n*)	Percentage (%)
Sex	Female	62	60.2
	Male	41	39.8
Age	18–20 years	4	3.9
	21–29 years	20	19.4
	30–39 years	35	34.0
	40–49 years	36	35.0
	50–59 years	8	7.8
Marital Status	Married	26	25.2
	Single	59	57.3
	Divorced	10	9.7
	Widowed	8	7.8
Educational Level	No education	21	20.4
	Primary	39	37.9
	Secondary	34	33.0
	Tertiary	9	8.7
Working Experience	≤three years	20	19.4
	4–10 years	39	37.9
	11–15 years	24	23.3
	16–20 years	14	13.6
	≥21 years	6	5.8

**Table 3:** Food safety training among participating food handlers.

Training Variable	Characteristic	Frequency (*n*)	Percentage (%)
Food safety training (*n* = 103)	Yes	16	15.5
	No	87	84.5
Venue of training (*n* = 16)	Oshakati	8	50.0
	Otjiwarongo	2	12.5
	Windhoek	6	37.5
The year of training (*n* = 16)	2018	1	6.3
	2019	1	6.3
	2020	3	18.8
	2021	4	25.0
	2022	7	43.8

**Table 4:** Association between food handlers’ food safety KAP and sociodemographic characteristics.

	Practices	Attitudes	Knowledge	Sex	Age	Marital Status	Education Level	Work Experience	Training
Practices	1	−0.745 **	0.745 **	0.051	−0.021	−0.144	0.113	0.172	0.038
Attitudes	−0.745 **	1	−1 **	−0.054	0.032	0.073	−0.126	−0.229 *	−0.02
Knowledge	0.745 **	−1 **	1	0.054	−0.032	−0.073	0.126	0.229 *	0.02
Sex	0.051	−0.054	0.054	1	0.052	−0.024	0.052	0.064	−0.02
Age	−0.021	0.032	−0.032	0.052	1	0.061	0.176	0.605 **	−0.035
Marital status	−0.144	0.073	−0.073	−0.024	0.061	1	0.094	0.052	−0.033
Educational level	0.113	−0.126	0.126	0.052	0.176	0.094	1	0.467 **	−0.096
Work experience	0.172	−0.229 *	0.229 *	0.064	0.605 **	0.052	0.467 **	1	−0.034
Training	0.038	−0.02	0.02	−0.02	−0.035	−0.033	−0.096	−0.034	1

Heat map of the Pearson correlation coefficient between each variable. Red colour denote a negative correlation while blue represents a positive correlation. Darker shades indicate stronger correlations, while lighter shades represent weaker correlations. * Significant correlation at the 0.05 level (2-tailed), ** Significant correlation at the 0.01 level (2-tailed).

**Table 5:** Multiple linear regression analysis of sociodemographic characteristics as predictors of food safety knowledge, attitudes, and practices among food handlers.

Independent Variables	Knowledge		Attitudes		Practices	
	β Coefficient	*p*-Value	β Coefficient	*p*-Value	β Coefficient	*p*-Value
Sex	0.041	0.674	−0.170	0.011	0.009	0.898
Age	−0.265	0.002 *	0.079	0.001	0.011	0.903
Marital Status	−0.075	0.440	−0.091	0.421	−0.092	0.180
Education Level	−0.003	0.975	−0.077	0.940	0.035	0.655
Work Experience	0.393	0.003 *	−0.162	0.000	−0.014	0.000 **
Training	0.022	0.818	−0.064	0.276	0.023	0.735

* Significance level: *p* < 0.05, ** Significance level: *p* < 0.001.

## Data Availability

Data are available upon request and within the prescripts of the Protection of Personal Information Act (POPIAct).

## Supplementary Materials

The following supporting information can be downloaded at: https://www.mdpi.com/article/10.3390/ijerph22040631/s1, File S1: QUESTIONNAIRE.
