# Supplementary material for: Food Safety in Informal Markets: How Knowledge and Attitudes Influence Vendor Practices in Namibia
Source: Int J Environ Res Public Health. 2025 Apr 17;22(4):631. doi: 10.3390/ijerph22040631 (PMC12026613; doi:10.3390/ijerph22040631)
Supplement: Supplementary file 1 [file ijerph-22-00631-s001.zip › ijerph-3535959-supplementary.pdf]

Supplementary Material S1: QUESTIONNAIRE

Good day, my name is Winnie Sheehama, and I am conducting a study entitled "**An assessment of food handlers' knowledge, attitudes and practices on food safety at Oshakati open market in Northern Namibia**" in partial fulfilment of a Master's Degree of Public Health at the University of Johannesburg.

Your response to this research will be anonymous, and all data will be confidential. The shared information will be reported as group data only and used exclusively for academic purposes. Therefore, you are encouraged to answer the questions as honestly as possible.

Instructions for completing the questionnaire: Please indicate your responses based on your knowledge, attitude, and practices by placing an "X" or a checkmark next to the corresponding response. Kindly fill out this questionnaire within a single day, and the researcher will collect the completed questionnaires once you have finished.

Thank you for your cooperation and support.

Participants Identity Code: ______________________ Date: _________________

**Section A: Demographic Information** *(Please complete all questions and mark with an (X) where appropriate)*			
A1	Gender	1=Male	
		2=Female	
A2	Please indicate your age range based on the options given.	1=≤20	
		2=21-29	
		3=30-39	
		4=40-49	
		5=50-59	
		6=≥60	
A3	Marital Status	1=Married	
		2=Single	
		3=Divorced	
		4=Widow/widowers	
A4	Indicate your highest qualification obtained.	1=No education	
		2=Primary education	
		3=Secondary education	
		4=Tertiary education	
A5	How long have you been working as a food handler?	1=≤3 years	
		2=4-10 years	
		3=11-15 years	
		4=16-20 years	
		5=≥21 years +	
A6	Are you formally trained in food safety?	1=Yes	
		2=No	

	If you answered 'YES' to A6 then continue with A6.1-A6.3. If you answered 'NO', then go to A7.		
A6.1	If **YES**, where was the training conducted?		
A6.2	If **YES**, when was the training conducted?	______/________/__________________	
A6.3	If **YES**, how did you acquire your knowledge of food safety?	1=Observation	
		2=Formal training	
		3=Other (*specify*)____________________	
A7	What is your nationality?	1= (*specify*)___________________	
A8	Are you registered with any authority as a food vendor?	1=Yes	
		2=No	
A9	Are you inspected by the health inspectors or other authorities?	1=Yes	
		2=No	

**Section B: Food Safety Knowledge of Food Handlers**			
**General knowledge**			
B1	Food safety knowledge by a food vendor will benefit consumers.	1=Yes	
		2=No	
		3=I don't know	
B2	Safe food handling should be an important part of your food vending business.	1=Yes	
		2=No	
		3=I don't know	
B3	Producing safe food is more important than producing tasty food.	1=Yes	
		2=No	
		3=I don't know	
B4	Street food vendors cannot safely handle food when handling money.	1=Yes	
		2=No	
		3=I don't know	
**Contamination and Transmission of Foodborne Diseases**			
B5	Good personal hygiene can prevent food contamination.	1=Yes	
		2=No	
		3=I don't know	
B6	Food handled by persons diseased or with wounded hands can cause food contamination.	1=Yes	
		2=No	
		3=I don't know	
B7	Contaminated foods always have some change in colour, smell or taste.	1=Yes	
		2=No	
		3=I don't know	
B8	It is important to check the expiry dates/best before food items.	1=Yes	
		2=No	
		3=I don't know	
B9	Contaminated food can cause foodborne illness to the consumer.	1=Yes	
		2=No	
		3=I don't know	
B10	Safe water can be seen by the way it looks.	1=Yes	
		2=No	

		3=I don't know	
B11	Wiping cloths can spread microorganisms.	1=Yes	
		2=No	
		3=I don't know	
B12	The same cutting board can be used for raw foods and cooked foods, provided it looks clean.	1=Yes	
		2=No	
		3=I don't know	
B13	Well-cooked foods are free of contamination.	1=Yes	
		2=No	
		3=I don't know	
B14	Raw and cooked foods should be stored separately to reduce the risk of food contamination.	1=Yes	
		2=No	
		3=I don't know	
B15	Proper cleaning and handling of food handling utensils reduce the risk of food contamination.	1=Yes	
		2=No	
		3=I don't know	
B16	Using hot water to clean utensils can reduce the risk of food contamination.	1=Yes	
		72=No	
		3=I don't know	
B17	It is important to change the water that you use to wash your utensils regularly.	1=Yes	
		2=No	
		3=I don't know	
**Temperature Control and Storage**			
B18	Reheating ready-to-eat food from the previous day for resale can contribute to food contamination.	1=Yes	
		2=No	
		3=I don't know	
B19	Defrosted foods can be refrozen.	1=Yes	
		2=No	
		3=I don't know	
B20	Food sold hot can be stored at ambient temperatures.	1=Yes	
		2=No	
		3=I don't know	
B21	It is important that every food vendor has heating facilities to store heated food products.	1=Yes	
		2=No	
		3=I don't know	
B22	It is important that every food vendor has chilling facilities to store food products that require chilling conditions.	1=Yes	
		2=No	
		3=I don't know	
B23	It is important that every food vendor has freezing facilities to store food products that require freezing conditions.	1=Yes	
		2=No	
		3=I don't know	
B24	In Namibia, the legally required core temperature of heated food products that	1=Yes	
		2=No	
		3=I don't know	

	are stored, transported, or displayed for sale is ≥60ºC.		
B25	In Namibia, the legally required core temperature of chilled food products that are stored, transported, or displayed for sale is ≤5ºC.	1=Yes	
		2=No	
		3=I don't know	
B26	In Namibia, the legally required core temperature of frozen food products that are stored, transported, or displayed for sale is ≥ -18ºC.	1=Yes	
		2=No	
		3=I don't know	
B27	It is important that every food vendor has a thermometer to monitor the temperature of food.	1=Yes	
		2=No	
		3=I don't know	
B28	Leftovers should be reheated to a minimum temperature of 75°C.	1=Yes	
		2=No	
		3=I don't know	
**Personal Hygiene**			
B29	Protective clothing reduces the risk of food contamination.	1=Yes	
		2=No	
		3=I don't know	
B30	Washing hands properly reduces the risk of food contamination.	1=Yes	
		2=No	
		3=I don't know	
B31	Washing hands with only water cannot clean enough.	1=Yes	
		2=No	
		3=I don't know	
B32	Hand washing is necessary for food vendors even when hands are not visibly dirty during continuous food handling	1=Yes	
		2=No	
		3=I don't know	
B33	When wearing gloves, you can handle cooked foods after handling raw meat.	1=Yes	
		2=No	
		3=I don't know	
B34	After using the toilet, you should always wash your hands with soap and running water.	1=Yes	
		2=No	
		3=I don't know	

**Section C: Food Safety Practices of Food Handlers**			
C1	Do you always wash your hands before handling unwrapped food?	1=Yes	
		2=No	
		3=Sometimes	
C2	Do you always wash your hands after handling unwrapped raw food?	1=Yes	
		2=No	
		3=Sometimes	
C3	What do you use to wash your hands?	1=Soap and running water	
		2=Running water only	
		3=Water in a container	
		4=Water in a container and soap	
C4		1=Yes	

	Do you wash your hands after going to the toilet?	2=No	
		3= Sometimes	
C5	Do you dry your hands after washing?	1=Yes	
		2=No	
		3= Sometimes	
C6	What do you use to dry your hands?	1=Paper towel	
		2=Dry cloth	
		3= Wet towel	
		4=Do not dry them	
		5=Other (*specify*)____________________	
C7	Do you always wear protective clothing when preparing or handling food?	1=Yes	
		2=No	
		3= Sometimes	
C8	When handling food, do you also handle money at the same time?	1=Yes	
		2=No	
		3= Sometimes	
C9	Do you handle food at this site while having diarrhoeal disease?	1=Yes	
		2=No	
		3= Sometimes	
C10	Do you wear jewelleries when handling food?	1=Yes	
		2=No	
		3= Sometimes	
C11	Do you rub your hands on the face, hair, arms etc. when handling food?	1=Yes	
		2=No	
		3= Sometimes	
C12	Do you keep your nails short and remove all adornments before handling food?	1=Yes	
		2=No	
		3= Sometimes	
C13	Do you blow air into a polythene bag before use?	1=Yes	
		2=No	
		3= Sometimes	
C14	Do you wash and sanitise the knife and chopping board after chopping raw chicken or meat or any other raw food?	1=Yes	
		2=No	
		3= Sometimes	
C15	Do you use detergent to clean utensils?	1=Yes	
		2=No	
		3= Sometimes	
C16	Where do you prepare your food?	1=Home	
		2=Market	
		3= Both	

C17	If you prepare your food at home how do you transport the food to the vending site? (*specify*)		
C18	Do you store raw and cooked food separately?	1=Yes	
		2=No	
		3=Sometimes	
C19	How do you store prepared food during vending hours?	1=Containers at ambient temperatures	
		2=Warmers	
		3=Coolers	
		4=Other(*specify*)____________________	
C20	How do you serve food items to consumers?	1=Into re-usable cup/plate	
		2=Paper plates	
		3=Plastic bags	
		4=Old newspaper	
		5= Other(*specify*)____________________	
C21	What do you do with the leftovers at the end of the day?	1=Consume at home	
		2=Store for use in the next day	
		3=Give it to needy people	
		4= Thrown away	
C22	Do you re-freeze defrosted food?	1=Yes	
		2=No	
		3= Sometimes	
C23	Do you use the same water to wash the utensils more than once	1=Yes	
		2=No	
		3=I don't know	

**Section D: Food Safety Attitudes of Food Handlers**			
D1	I am willing to learn about the basics of food safety.	1=Yes	
		2=No	
		3=I don't know	
D2	I wear a uniform during food preparation.	1=Yes	
		2=No	
		3=I don't know	
D3	I go for a medical health check every six months.	1=Yes	
		2=No	
		3=I don't know	
D4	I bath before and after coming to the market	1=Yes	
		2=No	
		3=I don't know	
D5	I keep long and painted fingernails.	1=Yes	
		2=No	
		3=I don't know	
D6	I smoke in the food preparation area.	1=Yes	
		2=No	
		3=I don't know	

D7	I overlap food dishes while serving food.	1=Yes	
		2=No	
		3=I don’t know	
D8	I take sick leave if I am sick.	1=Yes	
		2=No	
		3=I don’t know	
D9	I try my level best to always observe proper cleaning procedures.	1=Yes	
		2=No	
		3=I don’t know	
D10	Safe food handling is an important part of my job responsibilities.	1=Yes	
		2=No	
		3=I don’t know	
D11	I feel foodborne outbreaks are natural life event	1=Yes	
		2=No	
		3=I don’t know	

Thank you for your participation
